# A proof of concept study on reliability assessment of different metal foil length based piezoelectric sensor for electromechanical impedance techniques

**DOI:** 10.1038/s41598-023-49762-2

**Published:** 2024-01-06

**Authors:** Lukesh Parida, Sumedha Moharana, Romeu Vicente, Guilherme Ascensão

**Affiliations:** 1 Department of Civil Engineering, Shiv Nadar Institution of Eminence, Dadri, Uttar Pradesh 201314 India; 2 RISCO, Department of Civil Engineering, University of Aveiro, 3810-193 Aveiro, Portugal

**Keywords:** Civil engineering, Materials science, Structural materials, Techniques and instrumentation

## Abstract

Lead zirconate titanate (PZT) patches gained popularity in structural health monitoring (SHM) for its sensing and cost effective. However, a robust installation of PZT patches is challenging due to the often-complex geometry and non-accessibility of structural parts. For tubular structures, the curved surface can compromise the perfect bonding of PZT patches. To alleviate the above-mentioned challenges, the non-bonded and reusable configuration of sensor received considerable interest in the field of SHM. However, ensuring the repeatability and reproducibility of Electro-Mechanical Impedance (EMI) measurements is crucial to establish the reliability of these techniques. This work investigated the repeatability and reproducibility measures for one of non-bonded configuration of PZT patch i.e., Metal Foil Based Piezo Sensor (MFBPS). In addition, the concept, application, and suitability of MFBPS for impedance-based monitoring technique of Civil infrastructure are critically discussed. This study evaluates the effect of length of MFBPS on piezo coupled admittance signature. Also, this study evaluates repeatability and reproducibility of EMI measurements via statistical tools such as ANOVA and Gage R&R analysis. The statistical index CCDM was used to quantify the deviations of impedance signals. The overall result shows that the repeatability of the EMI measurements improves with a metal foil length of 500 mm. Overall, this investigation offers a useful point of reference for professionals and scholars to ensure the reliability of MFBPS for EMI techniques, a variant of piezoelectric sensor for SHM applications.

## Introduction

In the past few years, the field of structural health monitoring (SHM) has gathered substantial interest because of its ability to improve the safety and dependability of a wide range of structures. Monitoring the structural health of systems in a continuous manner is paramount to offer vital information that aids in averting catastrophic failures and enhancing operational efficiency. The benefits of SHM extend beyond safety and reliability, as it can also help reduce maintenance costs and extend the lifespan of structures. By providing a better understanding of the structural behavior of a system, engineers can optimize maintenance schedules, reduce unnecessary inspections, and avoid costly repairs. In this era of aging infrastructure and increasing demand for safety and sustainability, SHM is becoming an indispensable tool for engineers, architects, and asset managers to ensure the safety and functionality of our built environment^1^.

Among various SHM techniques developed over time, the Electro-Mechanical Impedance (EMI) techniques have become a highly promising approach for SHM due to their non-invasive, cost-effective, and real-time monitoring capabilities^2^. The EMI approach is an efficient method for detecting deterioration in SHM systems. This method uses piezoelectric transducers bonded or implanted into a structure. These transducers can transmit and receive acoustic signals, and by evaluating changes in electrical impedance at the PZT-structure interaction, one may efficiently monitor the structure’s health. When the host structure sustains damage, its mechanical properties change, resulting in changes in the impedance recorded by the piezo sensor. EMI techniques also identify and locate structural degradation by continually monitoring these impedance changes over time, offering vital insights into the observed structure’s integrity and safety. The method proposed by Bhalla and Soh^3^ involves measuring the admittance of a PZT patch that is bonded or embedded in a structure, and then using a set of equations to extract the complex admittance signature. Equation (1) shows the admittance signature of a PZT patch considering 2D interaction of PZT patch and structure.

1 $$\overline{Y} = G + Bj = 4\omega j\frac{{\mathop l\nolimits^{2} }}{h}\left[ {\overline{{\mathop \varepsilon \nolimits_{33}^{T} }} - \frac{{2\mathop d\nolimits_{31}^{2} \overline{{\mathop Y\nolimits^{E} }} }}{{\left( {1 - \mu } \right)}} + \frac{{2\mathop d\nolimits_{31}^{2} \overline{{\mathop Y\nolimits^{E} }} }}{{\left( {1 - \mu } \right)}}\left( {\frac{{\mathop Z\nolimits_{a,eff} }}{{\mathop Z\nolimits_{S,eff} + \mathop Z\nolimits_{a,eff} }}} \right)\overline{T} } \right]$$

where $\overline{Y}$ is the electrical impedance, h, l is the thickness and length of PZT patch, $\overline{{\mathop Y\nolimits^{E} }}$ is the complex youngs modulus, $\mathop Z\nolimits_{S,eff}$ and $\mathop Z\nolimits_{a,eff}$ is the effective impedance of structure and PZT, $\overline{T }=\frac{tankl}{kl}$, k is wave number, $\overline{{\mathop \varepsilon \nolimits_{33}^{T} }}$ is the complex electric permitivity, $\mathop d\nolimits_{31}^{{}}$ piezoelectric strain coefficient and µis the poissions ratio.

The EMI technique utilizes the inherent electrical properties of piezoelectric materials to monitor the mechanical impedance of a structure. The change in the impedance signature can be used to identify and locate damage, even at the very incipient level^4^. The method of EMI has been successfully demonstrated on diverse types of engineered constructions, including bridges, buildings, aircraft, and pipelines^5–7^. The non-destructive and non-invasive nature of the technique makes it particularly attractive for monitoring large and complex structures. Moreover, the low cost and ease of implementation of the EMI technique make it a practical solution for real-time monitoring of structures.

Although the EMI technique has shown promise for Structural Health Monitoring (SHM), there is still a need for additional research to address practical challenges associated with the method. A major hurdle in utilizing PZT patches is their fragility, which poses difficulties in affixing them to complex-shaped structures^8^. In situations where the component is frequently subjected to impact or utilized in an environment with high temperatures, directly bonding the patch onto the surface of the structure may not be feasible^9,10^. To overcome this challenge, alternative attachment methods need to be explored. Additionally, detecting damage can be challenging for certain composite materials like concrete, as the impedance signatures may not exhibit distinct peaks^11^. Moreover, traditional direct bonded piezo configurations have certain limitations that can make them unsuitable for some applications^12^. This has led to the development of non-bonded and reusable configurations that offer distinct advantages over direct bonded piezo configurations.

Non-bonded and reusable configurations offer distinct advantages over direct bonded piezo configurations in monitoring complex infrastructure systems. These configurations involve placing the piezoelectric sensor in contact with the structure being monitored using a non-permanent attachment method such as metal wire, magnets, clamps or adhesive tape^13,14^. This approach allows for quick and easy deployment of sensors and can be used in a wide range of applications. Furthermore, non-bonded configurations do not affect the mechanical properties of the structure, which helps to ensure the accuracy of the readings. Additionally, the non-bonded sensors can be removed and reused, making them a more cost-effective solution in the long term^15^. This allows for easy removal and repositioning of the sensor without damaging the structure or the sensor itself. Additionally, non-bonded sensors can be used multiple times, making them more cost-effective eventually^16^.

Na and Li^17^ proposes a novel approach that utilizes a steel wire coupled with a PZT element to overcome the limitations of the EMI technique for composite structures with complex surfaces. The investigation conducted a series of examinations to assess how wire lengths, wire diameters, and PZT sizes influence the impedance signature. Additionally, this study furnishes significant perspectives on utilizing the steel wire EMI technique to identify damage, debonding, and deterioration of the adhesive layer in composite structures. The study showed that the method can effectively detect damage and differentiate between different damage scenarios. The authors also highlighted the potential of the method for real-time monitoring of structures, as it can detect damage at an early stage.

Naskar and Bhalla^18^ present a novel approach for damage assessment of two-dimensional structures using metal-wire-based twin one-dimensional orthogonal array configuration of PZT patches. The authors highlight the importance of damage assessment in ensuring the reliability of structural systems.

Kaur et al.^19^ offer a cost effective solution using multi configurations to detect sensitivity and accuracy of damage detection. Parida and Moharana^20^ proposed multimodal piezo configurations such as metal wire, clamped, and rebar bonded to detect damage and loading effects of construction steel. This research underscores the challenges posed by the complex rebar surface and assesses the effectiveness of proposed configurations. Raju et al.^21^ present non-bonded piezo configurations to monitor corrosion-induced damage in pipeline structures. This study shows a potential replacement for typical bonded piezo sensors for corrosion detection and assessment in pipeline However, the repeatability of the impedance signatures is a critical factor that affects the reliability of the monitoring systems. In this regard, the study related to the repeatability of the impedance signatures at a suitable metal wire length has not been adequately explored. Repeatability and reproducibility study are essential for ensuring the reliability and validity of suitable metal wire length-based EMI techniques. Without these qualities, the technique may not be considered trustworthy or applicable in real-world situations. Automotive Industry Action Group (AIAG) defines repeatability as the variations in the measurement with the same operator, same part, or same instrument measured multiple times. This is frequently accomplished by taking repeated measurements on the same part and then evaluating the data to evaluate the repeatability of the measuring device. In contrast, reproducibility analyzes the variance in measurements when various operators or equipment are utilized. This is often assessed by measuring the same component using different operators or equipment. This work aimed to check the reliability and stabilization of the metal foil-based configuration signal using repeatability measurements.

Over the years, Measurement System Analysis (MSA) is an important statistical technique that plays a key role in ensuring quality control in various industries. This technique is used to evaluate the measurement systems that are used in production and manufacturing processes to ensure that they are accurate, reliable and precise^22^. The AIAG recommends a standardized approach to investigate measurement system (MS) to ensure that the data collected is reliable and can be used for making informed decisions. By investigating the MS, researchers can ensure that the data collected is accurate, reliable, and free from errors, thus increasing the validity and credibility of the research^23^. The importance of accurate and reliable measurements in decision-making cannot be overstated. MSA evaluating the capability of a measurement device or gauge to produce reliable and accurate data that can be used for making informed decisions^24^. It involves quantifying the system’s ability to provide reliable data.

Several statistical techniques have been developed to evaluate the repeatability and reproducibility of a measurement system, including analysis of variance (ANOVA) and the use of control charts^25^. ANOVA and control charts are powerful statistical tools used to evaluate the reliability of a measurement system, and their use can significantly improve the quality and efficiency of measurement processes^26^. ANOVA has several advantages over other statistical tests, including its ability to manage multiple groups simultaneously and its ability to account for variation within and between groups. ANOVA has been applied in steel and aluminum turning to conduct research on the impact of cutting parameters on surface roughness and cutting force^27^. ANOVA has also been used in quality control of food to evaluate the effects of different processing conditions on the quality of the final product^28^, and in the automotive industry to optimize the performance of engines by identify factors affecting fuel consumption^29^. In orthodontics, ANOVA has been used to evaluate the effectiveness of different orthodontic treatments on tooth movement and to compare the results of different treatment modalities^30^. Veit^31^ asserts that variation is an intrinsic characteristic of any process, permeating various aspects such as material properties, equipment conditions, and inspection methods, ultimately contributing to the occurrence of defects. Nomelini^32^ highlights the methods utilized for analyzing the component variability within a system, such as the techniques of R&R study.

The use of statistical techniques to assess the repeatability of measurement systems is essential in ensuring accurate and reliable results. Analysis of variance (ANOVA) and control charts are powerful statistical tools used to evaluate the repeatability and reproducibility of measurement systems. ANOVA is particularly advantageous in handling multiple groups simultaneously and accounting for variation within and between groups. It has been widely applied across various industries such as steel and aluminum turning, quality control of food, automotive industry, and orthodontics. Moreover, the variation is an intrinsic characteristic of any process that ultimately contributes to the occurrence of defects. R&R studies are one such technique that has been used to analyze component variability within a system. In the context of estimating metal foil length-based electro-mechanical impedance measurements, R&R studies are crucial in ensuring reliable, consistent, and replicable results. By examining the data set from the experiment, researchers can evaluate the accuracy of the measurements and determine any sources of variability that may impact the results. Thus, this study highlights the importance of conducting R&R studies in experimental design to ensure the integrity of the findings.

This paper presents a comprehensive investigation of different lengths of metal foil-based piezoelectric transducer (PZT) configurations attached to construction steel rebar for realistic structural health monitoring (SHM) applications. The study evaluates the repeatability and reproducibility measures of PZT sensors with varying lengths of metal foil, ranging from 100 to 500 mm, and compares their accuracy and reliability. This study also explores the potential of non-bonded and reusable configurations of PZT sensors, which is significant for complex structural geometries. The ANOVA-based analysis is employed to evaluate the contribution of several factors towards the potential of these configurations for impedance-based SHM. The results demonstrate that the 500 mm length metal foil performs better in terms of sensor stabilization and signal repeatability, with high accuracy indicated by the gauge R&R study. The findings of this study contribute to the ongoing research in SHM and provide valuable insights for the development of reliable and efficient EMI sensors for practical applications. The novelty of the study is as follows.

- The novelty of the research focused on investigating MFBPS configurations for effective sensor design and implementation.
- The application of probabilistic methods such as ANOVA and Gage R&R analysis, using statistical index CCDM to evaluate suitable metal foil Length Effects for impedance measurement, provides a new quantitative dimension to the investigation.
- Reliability of Metal foil based piezo sensor measurements utilizing statistical methods such as ANOVA and Gage R&R analysis.
- To investigate the Optimal Metal Foil Length on piezo-coupled admittance signatures for SHM applications.

## Experimental details

This study investigated the repeatability and reproducibility of Metal Foil Based Piezo Sensor (MFBPS) with five different length variation and its effect of coupled admittance signature. The proof of concept was established through a series of experiments conducted on three identical steel specimens of length 600 mm and diameter 16 mm.

### Specimen preparation

**Figure 1:**
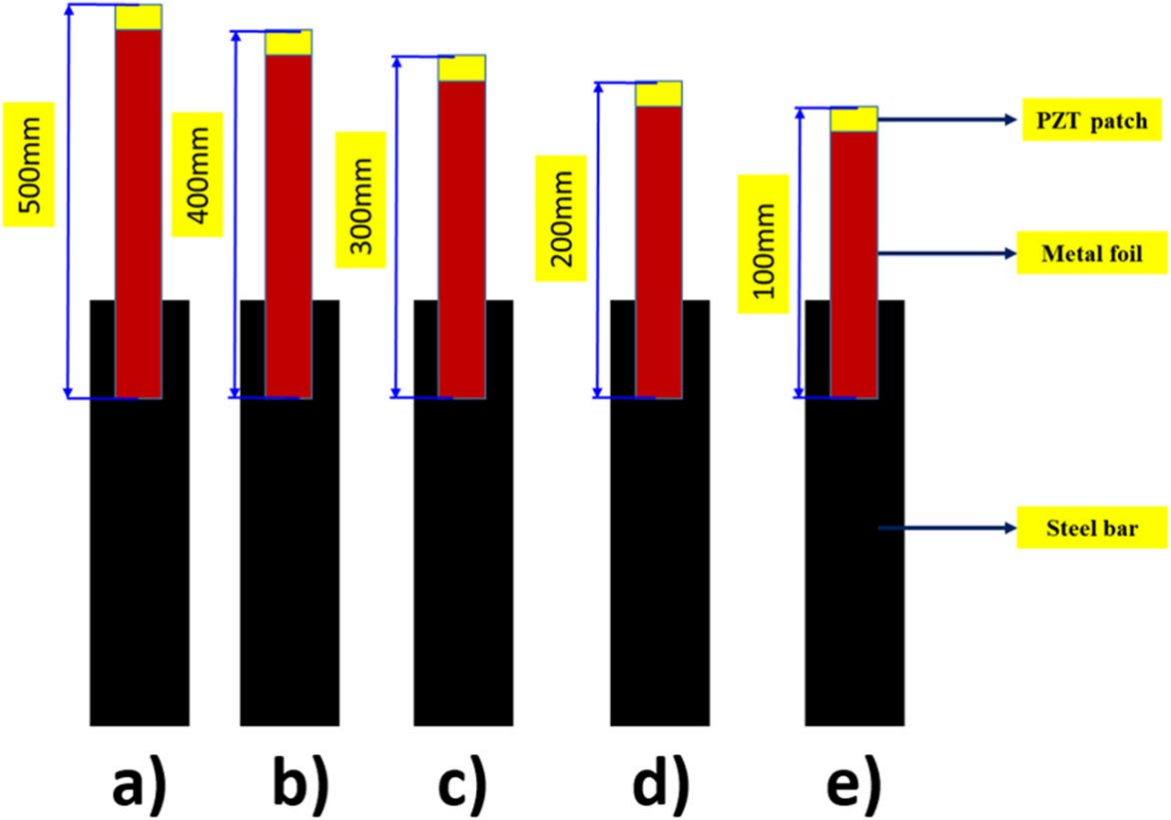
Schematic diagram for different metal foil attached to steel rebar (**a**) 500 mm (**b**) 400 mm (**c**) 300 mm (**d**) 200 mm (**e**) 100 mm.

**Figure 2:**
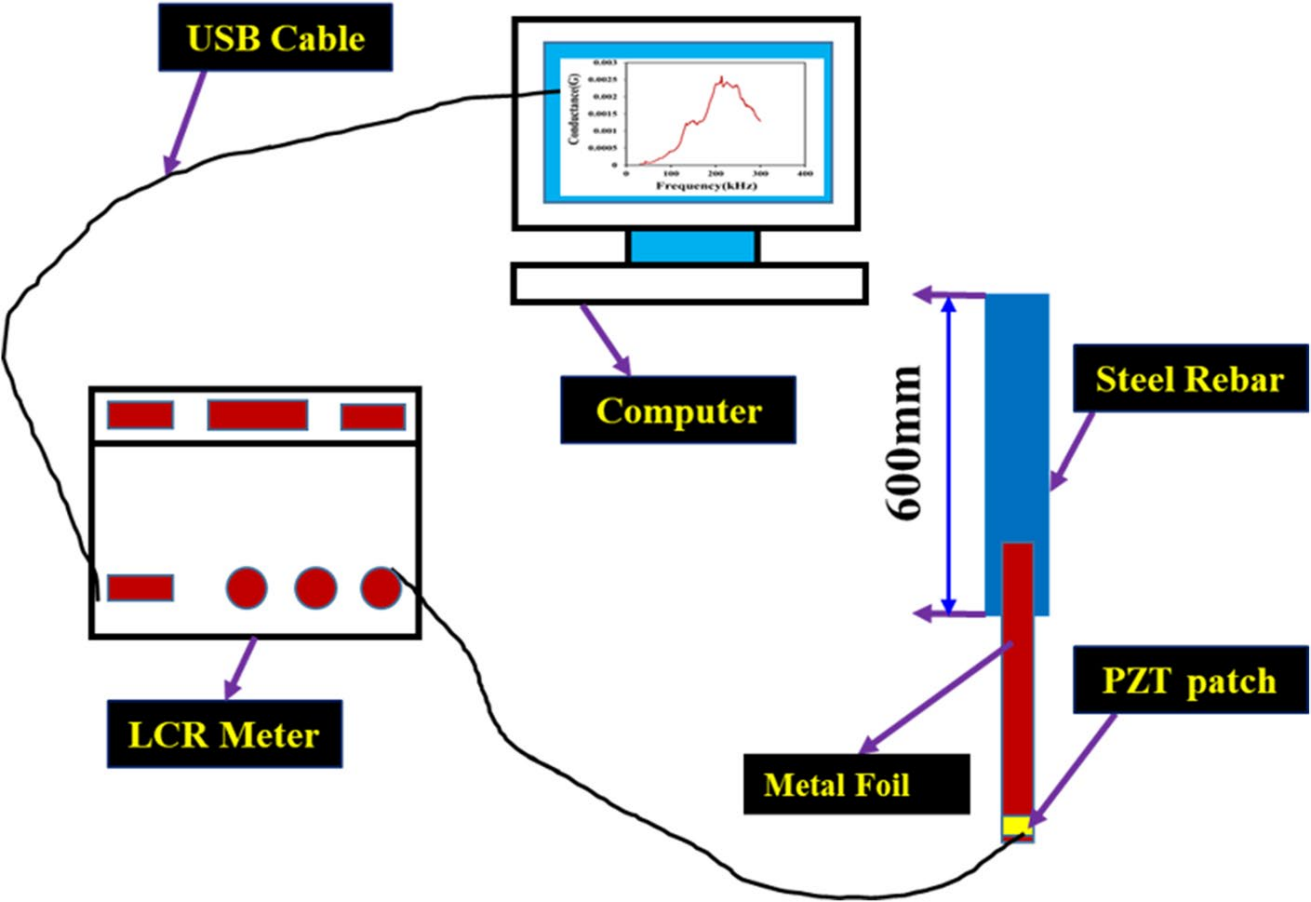
Schematic diagram for metal foil-based EMI measurement system.

The five metal foil variants were prepared with different lengths of 100 mm, 200 mm, 300 mm, 400 mm, and 500 mm with careful handling to avoid any damage or bending. The width and thickness of metal wire (MW) were 12 mm and 1 mm, respectively. Figures 1 and 2 illustrate the experimental setup where a PZT patch was bonded to one end of an aluminum foil, while the other end was inserted up to 50 mm from the top surface of the reinforced steel sample. For this study, five different aluminum foil length-based EMI techniques were used on three different constructional steel samples of length 600 mm and diameter 16 mm as per IS 432-1982. The PZT patches used in the study were square-shaped with dimensions of 10 × 10 × 0.3 mm and adhered to grade PIC 151 (manufactured by PI Ceramic, 2017). Despite the compromise in sensitivity of the piezo-coupled structural interaction in this configuration, the MFBPS variant proves to be a viable solution in scenarios where the traditional EMI technique cannot be implemented, such as in complex junctions of rail bridge girders or pier caps. The MFBPS technique offers several advantages, one of which is the ability to maintain resonance at specific frequencies regardless of the host structure’s nature. This is particularly beneficial in materials with high damping, such as concrete and certain ceramics, where mechanical resonance can be a concern. By attaching the PZT patches at the end of the metal wire, the MFBPS technique ensures that resonance is effectively maintained, resulting in a higher degree of reusability for this configuration of piezo sensor.

**Figure 3:**
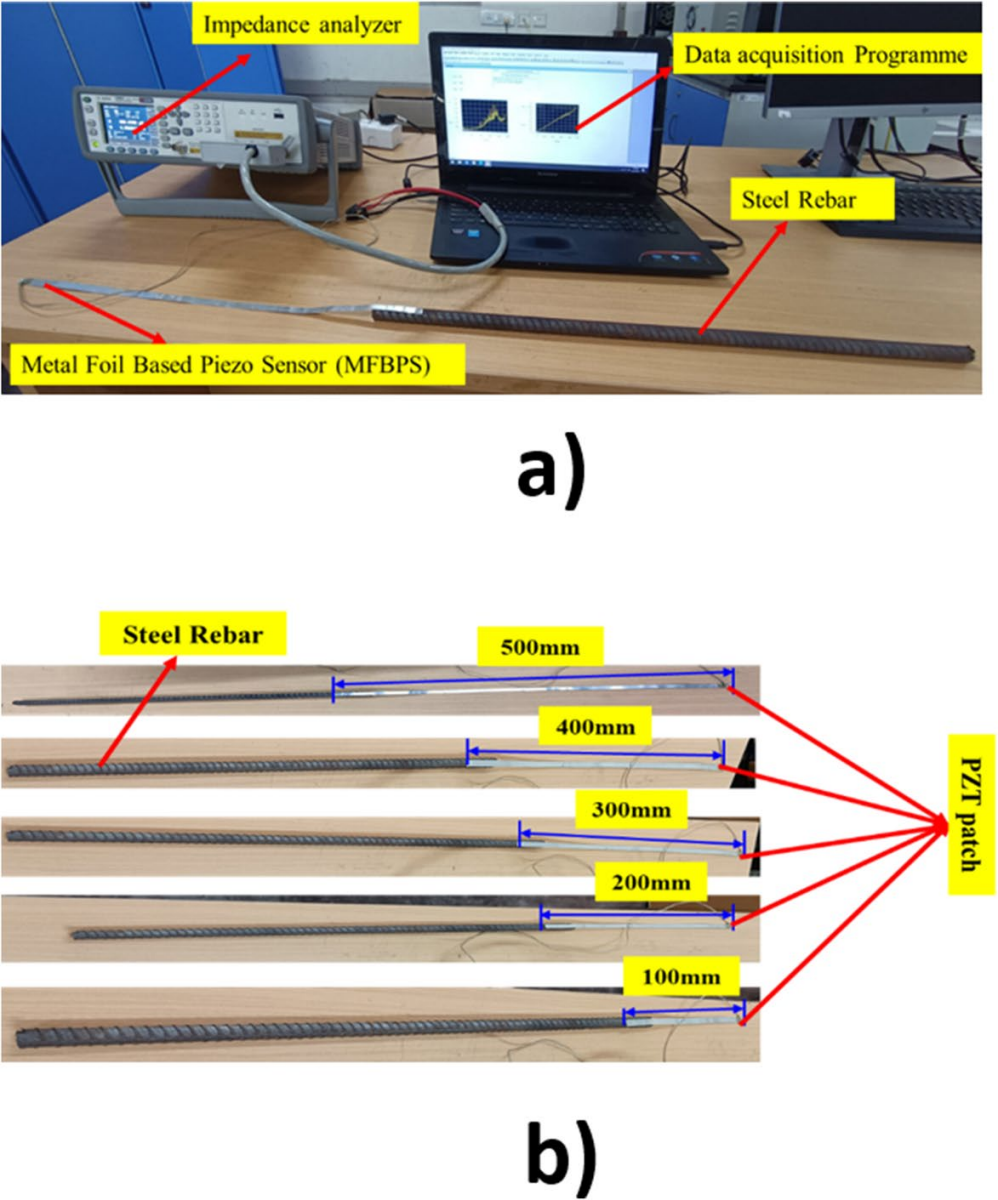
Experimental setup for (**a**) MFBPS measurement system (**b**) length of the different metal foil attached to steel rebars.

The application of a PZT patch at the end of a metal foil allows for easy removal of the foil from the embedded part, making it possible to reuse it for monitoring purposes in other structures. This unique flexibility is not present in previously demonstrated reusable sensors, which required partial or full embedment, resulting in potential damage to the structure when enabling it to reuse for monitoring purposes. The PZT patch of dimension was first attached to the end of the aluminum foil using epoxy adhesive uniformly and cured for 24 h at room temperature, i.e., 27 °C. After the curing process, the aluminum foil was then attached to the surface of the steel using epoxy adhesive. The epoxy glue was applied uniformly to the steel bar surface, resulting in a homogenous coating with no excessive accumulation. Controlled pressure was given to the patch to ensure proper bonding while the adhesive remained liquid. The PZT patches were protected by adding thin coating of adhesives. The electrodes were then connected to the PZT using conductive wires to ensure proper signal transmission. Figure 3 shows the detailed schematic diagram of the various PZT attached metal wire length attached to the construction steel sample.

### Experimental setup

The complete experimental setup, the steel bar instrumented with Metal Foil Based Piezo Sensor (MFBPS) and impedance analyzer, and excited with a frequency range of 30–300 kHz. The coupled admittance signature was obtained for steel sample with varying length of metal foil, Fig. 3.

### Repeatability and reproducibility measures

For sensor signal stabilization, most of experiment studied include the repeatability of signature with barely check with overlaps of peak conductance value. The plot of statistical indices do signify the overall change pattern but to assess the robustness of any experiment reproducibility and repeatability (R&R) is very important tool for statistically reliable. ANOVA model is commonly used to identify and eliminate sources of variability of intended experimental design. MSA is a crucial for analyzing the variation present in each type of inspection, measurement, and test equipment for any experimental design. While a surface bonded piezo Configuration has been considered for better actuation and sensing for structural information identification the risk and reliability non-bonded and reusable configuration is always a challenge. Hence, measuring the repeatability and reproducibility of experimental yields consistent and quality. The obtained results from ANOVA can be used to draw accurate conclusions. In this paper, the repeatability of the EMI observations was repetitively recorded for 20 times for each metal foil length, bonded to steel sample. In this study, the researchers investigated the suitability of non-bonded configuration PZT patch i.e., Metal Foil Based Piezo Sensor (MFBPS) for ensuring its repeatability and reproducibility as par with surface bonded configuration. In addition, the variation length of metal wire and its effect on overall piezo coupled admittance signature was also studied in this work. The three laboratory-sized reinforced steel bars equipped with metal foil based PZT patches were utilized to acquire conductance signatures for a specific time period, ensuring sensor readings and good repeatability. The healthy signatures were obtained for five different length i.e. 100 mm, 200 mm, 300 mm, 400 mm, and 500 mm; ranging from 30 to 300 kHz frequency, at 27 °C. This was done without imposing any structural load on steel. The reproducibility of the EMI measurements was assessed by repeating the measurements on specimens using different operators for each metal foil length. The obtained result demonstrates the importance of selecting the appropriate foil length for selected non-bonded and reusable piezo configuration i.e., MFBPS for impedance based SHM.

## Results and discussion

### Sensor stabilization

**Figure 4:**
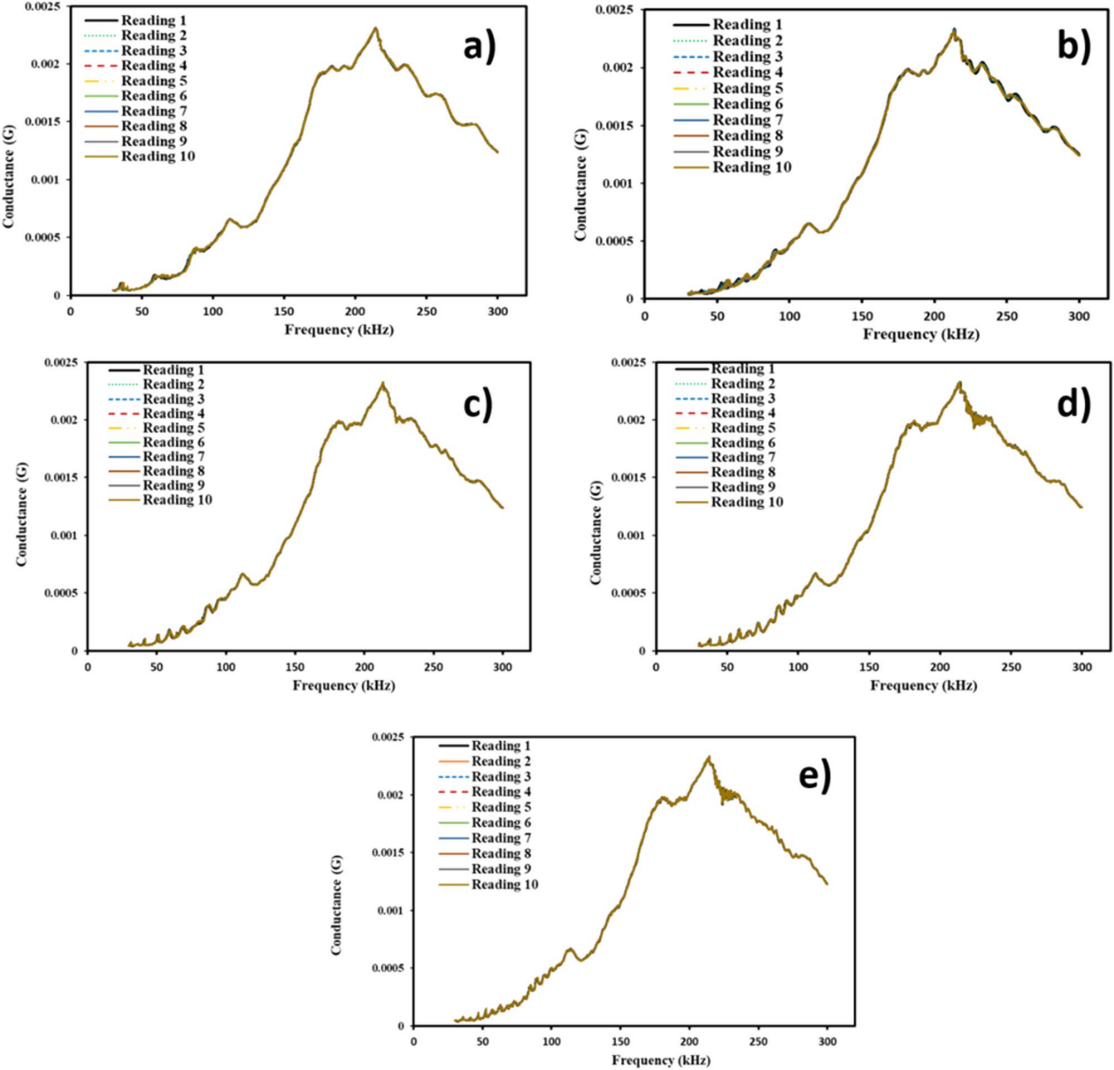
Conductance vs. frequency for metal foil lengths of (**a**) 100 mm (**b**) 200 mm (**c**) 300 mm (**d**) 400 mm (**e**) 500 mm.

Infrastructure systems can degrade over time, which can have adverse effect on their reliability and lifespan. The signal stabilization of pre-installed sensor is crucial to ensuring consistent performance in such situations. The stability of the sensors is essential for the measurement of EMI signals because of baseline approach for damage quantification. Sensor stabilization is a critical aspect of maintaining infrastructure systems and ensuring their optimal performance. As sensors are sensitive to external factors such as temperature, humidity, and noise, the electrical signal attenuation changes very rapidly, hence the inaccurate sensing can lead to inaccurate measurement of the EMI signals. Therefore, it is necessary to stabilize the sensors to minimize the effects of external factors and ensure that the impedance of the sensors remains constant throughout the measurement process. To achieve baseline signatures required for the EMI technique, sensor stabilization of a metal foil-based configuration of steel samples is necessary. Figure 4a–e shows repeatability of the signatures of the different MFBPS. It shows a consistent pattern, indicating that the bonding process is stable and reliable, very crucial indicator for the performance and longevity of the PZT sensor.

### Statistical indices

In impedance based SHM for damage quantification, many statistical indices are being used i.e., root-mean-square deviation (RMSD), mean absolute percentage deviation (MAPD), and correlation coefficient deviation metric (CCDM). The CCDM^5^ is commonly used to evaluate changes in signal measurement due to any structural anomaly or any electrical fluctuation. The CCDM index for the piezo-coupled signatures can be expressed by Eq. (2) ^33^.

2 $${\text{CCDM}}=1-\frac{\mathrm{cov }[Re\left({G}_{j}^{1}\right){,Re(G}_{j}^{i})]}{{\upsigma }_{1}{\upsigma }_{2}}$$

where $cov$ is the covariance between the ${G}_{j}^{1}$ and ${G}_{j}^{2}$. ${\upsigma }_{1}$ and ${\upsigma }_{2}$ represent the standard deviations of each signature. This metric assesses the level of concordance between the measurements.

**Figure 5:**
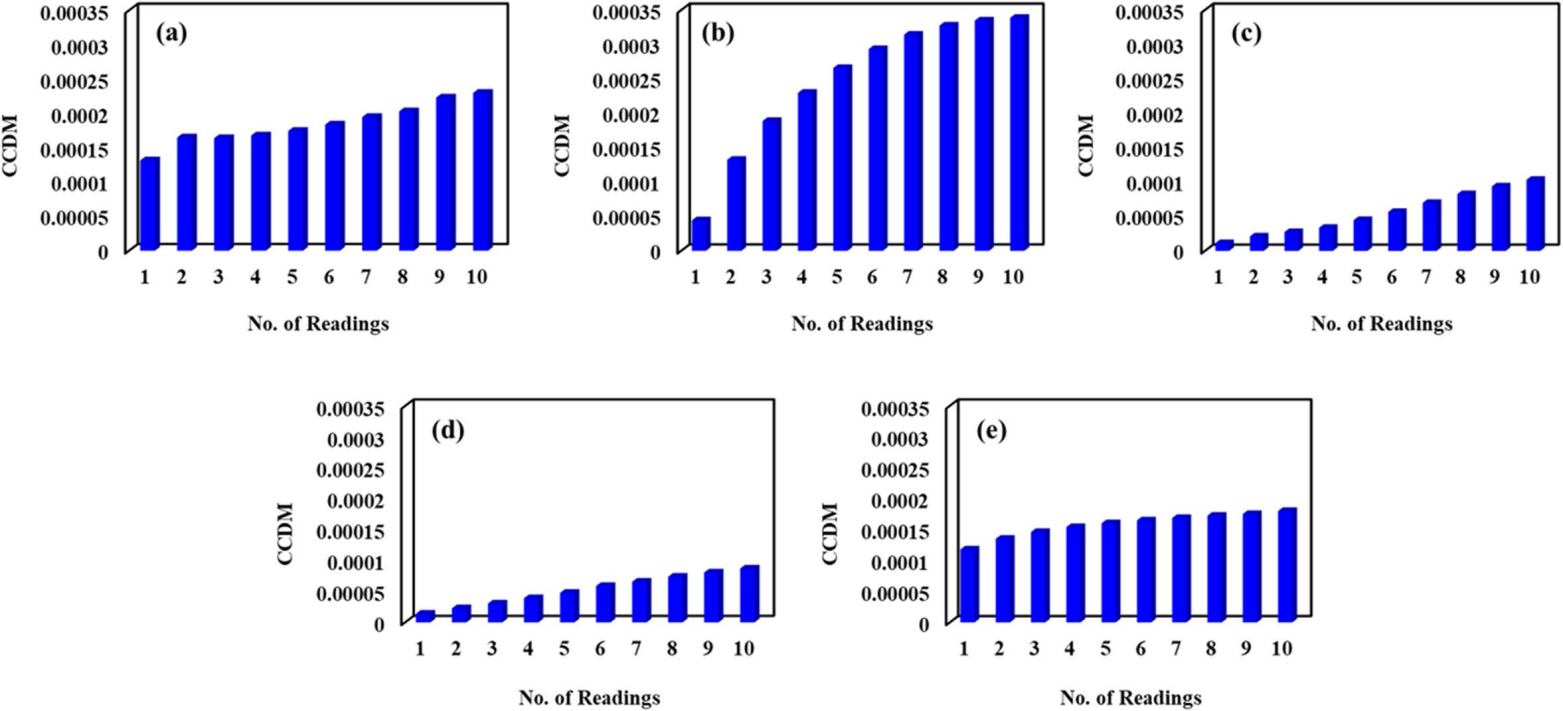
Statistical variations at different metal foil length.

Figure 5a–e shows the CCDM index variation for various metal foil lengths. For every length, after having repetitive signature, CCDM indices were plot and to study the preliminary variation between them. The repeatability of signature for different metal foil lengths and its trends in CCDM values show diverse patterns. The trend shows a high degree of consistency for the 500 mm metal foil length and is almost constant (Fig. 5e). Contrarily, the CCDM values for other metal foil length configurations, such as 100 mm, 200 mm, 300 mm, and 400 mm, show a rising tendency over time, lacking consistency as compared to the 500 mm configuration. Figure 5a–d graphically illustrates these observations. As this damage indices very crude towards signature repeatability, which encourages the authors to do in depth investigation as R &R study.

### Repeatability and reproducibility (R&R)

This section covers proposed R&R techniques for the repetitive EMI signal measured through impedance signature. This approach allowed for a more comprehensive understanding of the factors contributing to the variation in the results. In any system, the variability present can be attributed to two main factors: the inherent variability of the underlying process and the variability of the measurement system used to evaluate it, is illustrated in Eq. (3) ^34^.

3 $${\sigma }_{Total}^{2}={\sigma }_{Process}^{2}+{\sigma }_{Measurement}^{2}$$

The verification of the measurement system is an essential step in determining the true variability of a process under investigation. This verification process helps to identify and eliminate any sources of measurement error, thus providing accurate and precise data for further analysis^35,36^. Reproducibility and repeatability are the two key elements that contribute to the measurement system variance is outlined in Eq. (4) ^34^.

4 $${\sigma }_{Measurement}^{2}={\sigma }_{Repeatability}^{2}+{\sigma }_{Reproducibility}^{2}$$

Assessing the accuracy and precision of measurement processes requires careful consideration of two essential parameters—repeatability and reproducibility. Repeatability can be evaluated by measuring the same part multiple times with the same measurement device and recording the results. This practice enables the operator to assess the consistency of the measurements, making it a vital aspect of quality control in any measurement process. The variance of these measurements represents the degree of repeatability. To ensure reproducibility, it is necessary to have multiple operators measure the same part using the same measurement device. This enables the calculation of an average based on the measurements taken by different individuals^37,38^. The range method, average and range method, and analysis of variance method are the three main techniques for performing Gauge R&R. The range and average range technique approximate measurement variability but does not compute repeatability and reproducibility of the measurement equipment individually. The widely used and precise analysis of variance (ANOVA) approach is used to determine the repeatability and reproducibility of a measuring system. Additionally, it measures the variability of how the parts and the operator interact.

Repeatability and reproducibility measures are essential for evaluating the reliability of the results obtained from a model of analysis of variance (ANOVA). Here the author used CCDM index to study the repeatability and reproducibility measures for different metal wire lengths. Variance based ANOVA analysis was carried out using CCDM metrics from each foil length with 20 repetitions using MINITAB software. This analysis helps to study the efficiency and capability of metal foil based PZT patches. The study considers a randomized design with two factors i.e., two operators and five parts/metal foil length with 20 repetitions on each length. The repeatability measurement is equally shared by two operators. In the analysis of the variability of the CCDM metric, the authors utilized the analysis of variance technique to estimate the respective percentages of contribution variance, study variance and number of distinct categories (NDC) in relation to the total variation of the data (Table 1). Contribution variance percentages relate to the amount of variance percentage and measure how much each component contributes to the overall variance in a dataset. This parameter was used to identify the most influential variables in a dataset. Moreover, Study variance represents the variation in the results of the analysis. It shows the degree to which the data points deviate from the mean value. Different sample features and measurement fluctuations can all contribute to variability. NDC represents the number of distinct groups or categories in a dataset. This critical variable was utilized to analyses the distribution of data in categorical variables. The Automotive Industry Action Group (AIAG)^39^ has provided the guidelines for assessing measurement systems using % contribution variance, study variance and NDC (Table 2).

**Table 1:** CCDM metrics of estimated variance contribution percentages to the total variation in the R&R study.

MW length (mm)	Total gage R&R		NDC
	% Contribution variance	%Study variance	
100	1.92	13.87	10
200	0.40	6.36	22
300	39.93	63.19	1
400	0.83	9.11	15
500	0.13	3.54	39

**Table 2:** Automotive Industry Action Group (AIAG) guidelines^19^.

Source	Decision rule		
	Adequate measuring system	Acceptable based on application, cost of repair and device, other factors	Unacceptable and should be improved
% contribution variance	< 1%	> = 1% & < = 9%	> 9%
% Study variance	< 10%	> = 10% & < = 30%	> 30%
Number of Distinct Categories (NDC)	> = 5		< 5

It can be observed that the contribution % of overall R&R values is lower in the case of 500 mm metal foil length i.e. 0.13. This indicates that the 500 mm length possesses an acceptable measurement system for variance analysis. Typically, experiments yield better outcomes when R&R values are lower^40^. Moreover, 200 mm and 400 mm metal foil length show acceptable measurement system, but the % contribution variance is lower in 500 mm. It has been found from the research, a suitable measurement system that meets the AIAG group guidelines, requires less than 10% variance. On this account, the study variance found to be only 3.54% for a 500 mm metal foil length. While both 200 mm and 400 mm metal foil lengths meet the AIAG guidelines for an adequate measuring system, but the variance is much lower for the 500 mm length, making it more suitable for impedance signal measurements. On the other hand, 100 mm and 300 mm metal foil lengths have unacceptable variance contributions and require improvement. The NDC for the 500 mm length is higher, indicating an acceptable measuring system compared to other metal foil lengths^40^. However, the NDC value is 1 for the 300 mm length, indicating an inadequate measurement system.

**Figure 6:**
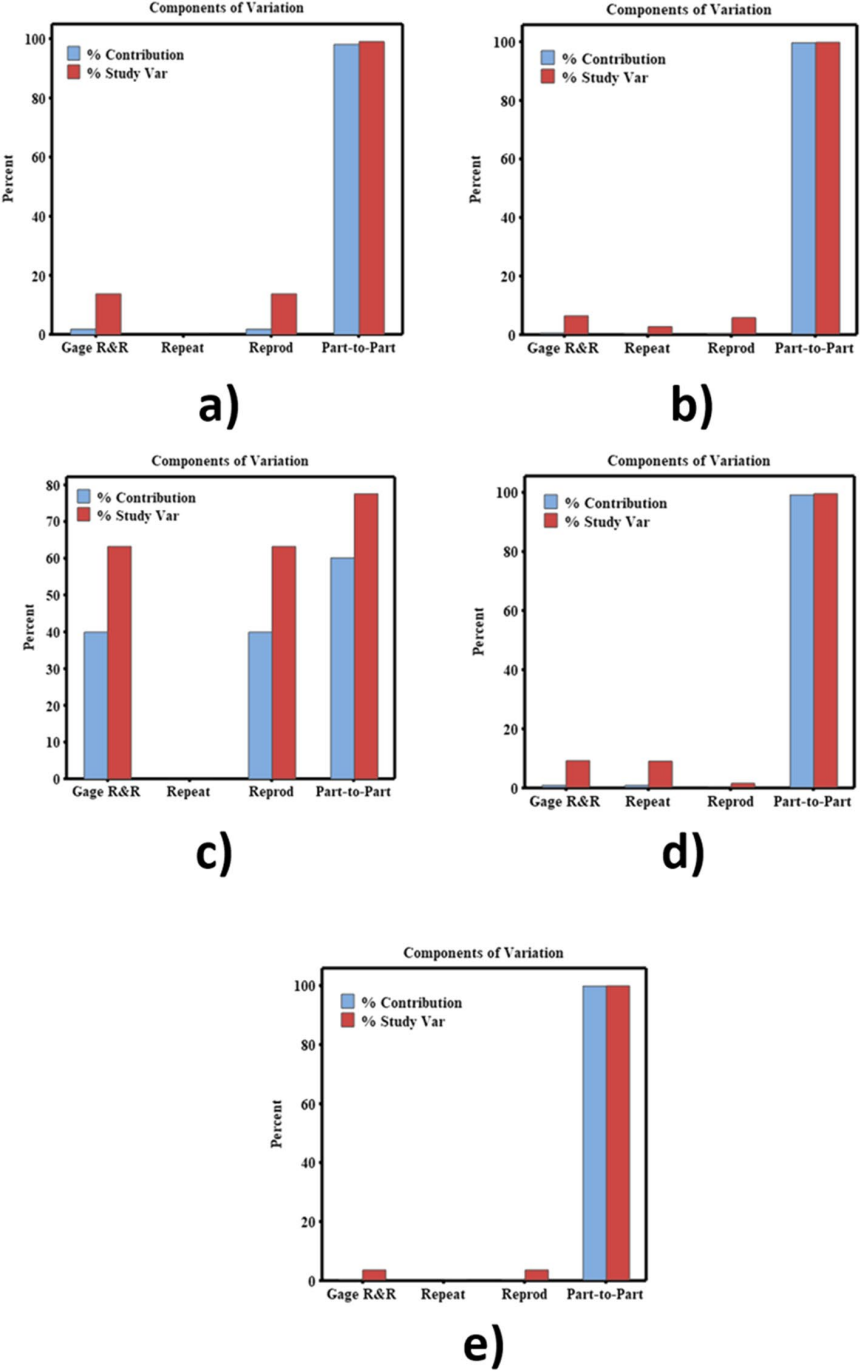
Components of variations at different metal foil length (**a**) 100 mm (**b**) 200 mm (**c**) 300 mm (**d**) 400 mm (**e**) 500 mm.

**Figure 7:**
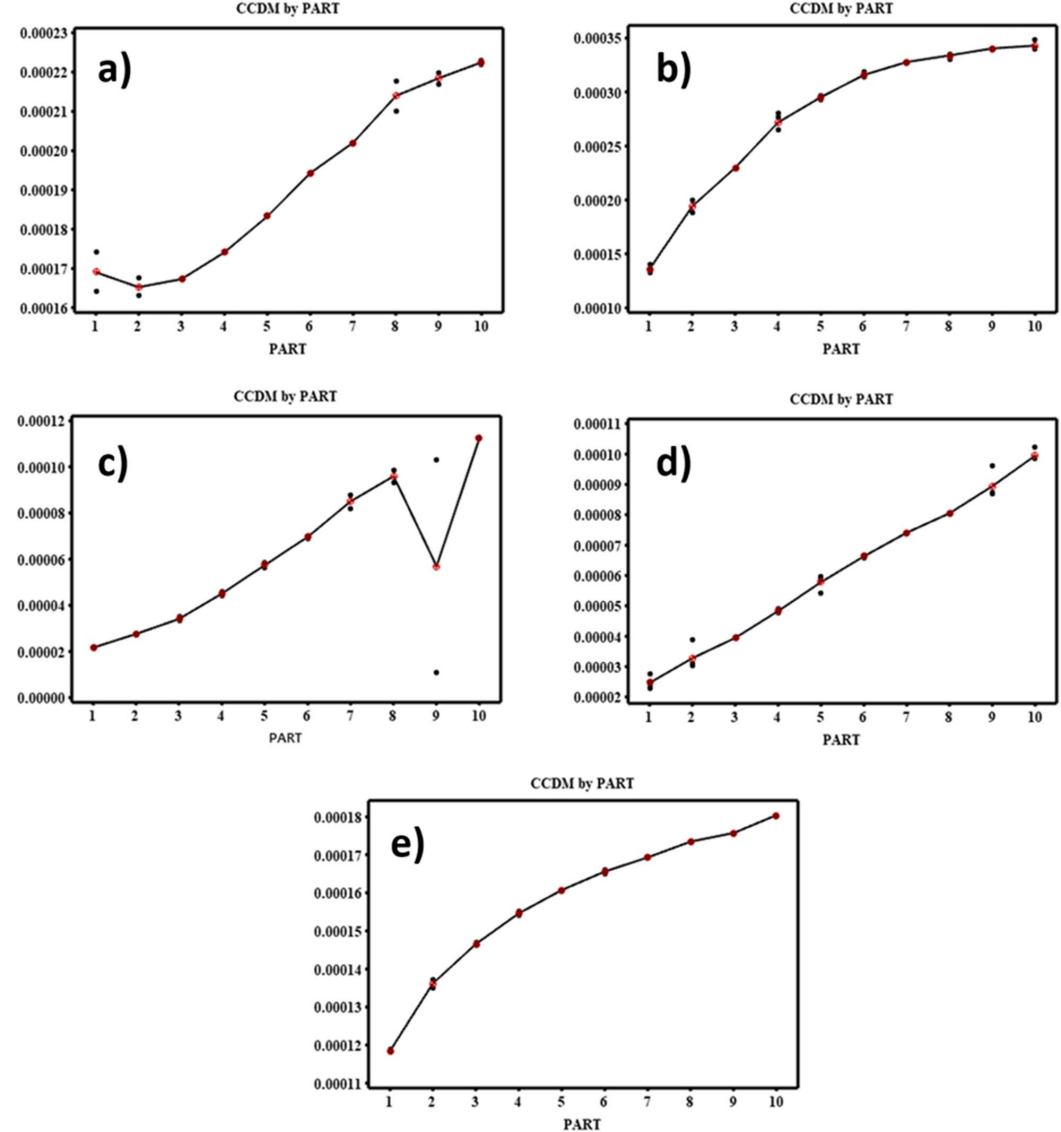
Variations of CCDM by parts at different metal foil length (**a**) 100 mm (**b**) 200 mm (**c**) 300 mm (**d**) 400 mm (**e**) 500 mm.

**Figure 8:**
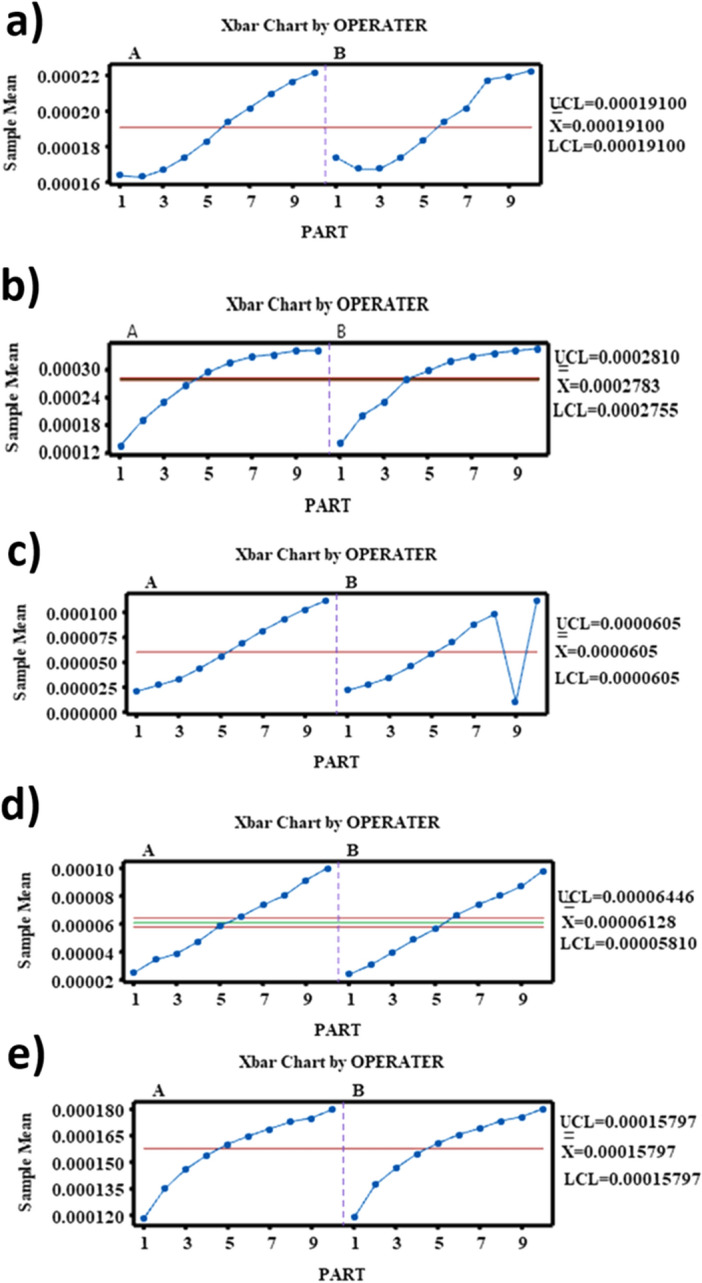
Mean chart by operator at different metal foil length (**a**) 100 mm (**b**) 200 mm (**c**) 300 mm (**d**) 400 mm (**e**) 500 mm.

Figure 6 shows the component variations of CCDM metrics for different metal foil lengths. It has been seen that larger variance caused by the measurement system indicates an adequate measuring system in case of 500 mm metal foil length. A reliable measurement system should have a smaller variance caused by the measurement system when compared to the variance between individual parts. If the part-part variations, bars in the column are high, it indicates a stable piezo-signal measurement system. Figure 7 shows the variations of CCDM by parts for different length of metal foils. This study presents the findings of the measurements and averages obtained for each part. The goal is to have the data collected for each part overlap in the chart. However, Fig. 7 shows the measurement variance of the parts remains consistent for a metal foil length of 500 mm. Moreover, in the case of other metal foil configurations the results were inconsistent. These findings suggest that further investigation is needed to identify the sources of measurement error and to improve the accuracy and reliability of the measurements in case of 100 mm, 200 mm, 300 mm, 400 mm length. Figure 8. indicates the $\overline{{\text{x}} }$ chart of the operators to know the results of the variance due to metal foil length or the measurement process itself. From this chart, for the best experimental design and its outcome, the higher variance in the piezo signal Configuration in not acceptable but rather the process (hence the frequency sweep calibration, i.e., LCR meter) itself should be the cause faulty results and robust measuring system. Measurements being within control limits (i.e., upper control limit (UCL) and lower control limit (LCL)) shows that an incorrect or faulty measurement system was employed. In Fig. 8, the mean chart shows the acceptable measuring system in case of 500 mm metal foil length. The variation in variance is caused due to measurement of metal foil length.

## Conclusions

This paper comprises repeatability & reproducibility study of different metal foil length based PZT attached to construction steel rebar. In this study, we investigated the repeatability and reproducibility measures for different aluminum foil length-based electro-mechanical impedance techniques. The aim was to demonstrate a statistical proof of concept for the effectiveness, and reliability of the piezo configuration in realistic structural applications. Through the experimental results and statistical investigation, it is found that the sensor stabilization and signal repeatability of 500 mm length metal foil performs better than 100 mm,200 mm, 300 mm & 400 mm. For 500 mm metal foil length, the gauge R&R study shows very promising results with greater accuracy. In total gauge R&R study, the parametric variation of sensor (for non-bonded and reusable) length has shown equal accuracy towards regular bonding process (i.e., surface bonded). The length variation of metal is significant for non-contact based sensor integrated SHM, where complex structural geometry becomes a constraint for infrastructure health management. With repetitive measurement, the piezo coupled signature acquired with operator and one equipment. The measurement variance was categorially evaluated for statistical matrix CCDM, derived from piezo driven signals. From ANOVA based study, the measurement variances i.e., % contribution of study part (i.e., length of metal wire) and contribution variance (i.e., piezo driven signal attached to metal with varying length) towards the potential evaluation and usage for reusable and non-bonded configuration of sensor for piezo impedance structural health monitoring. In overall, this study demonstrates the potential of aluminum foil length-based electro-mechanical impedance techniques as a reliable and effective tool for monitoring complex infrastructures. The proposed can add to the continuing research in SHM and provides insights for the development of reliable and efficient EMI sensors for practical applications where is not feasible to place PZT patches directly to the host structure.

## Data Availability

All data generated or analyzed during this study are included in this published article.
